# Exploring European Heavy Goods Vehicle Crashes Using a Three-Level Analysis of Crash Data

**DOI:** 10.3390/ijerph19020663

**Published:** 2022-01-07

**Authors:** Ron Schindler, Michael Jänsch, András Bálint, Heiko Johannsen

**Affiliations:** 1 Department of Mechanics and Maritime Sciences, Chalmers University of Technology, 41756 Göteborg, Sweden; andras.balint@chalmers.se; 2 Accident Research Unit, Medizinische Hochschule Hannover, 30625 Hannover, Germany; jaensch.michael@mh-hannover.de (M.J.); johannsen.heiko@mh-hannover.de (H.J.)

**Keywords:** long-haul truck, crash scenarios, GIDAS, CARE, crash causation, European national crash data

## Abstract

Heavy goods vehicles (HGVs) are involved in 4.5% of police-reported road crashes in Europe and 14.2% of fatal road crashes. Active and passive safety systems can help to prevent crashes or mitigate the consequences but need detailed scenarios based on analysis of region-specific data to be designed effectively; however, a sufficiently detailed overview focusing on long-haul trucks is not available for Europe. The aim of this paper is to give a comprehensive and up-to-date analysis of crashes in the European Union that involve HGVs weighing 16 tons or more (16 t+). The identification of the most critical scenarios and their characteristics is based on a three-level analysis, as follows. Crash statistics based on data from the Community Database on Accidents on the Roads in Europe (CARE) provide a general overview of crashes involving HGVs. These results are complemented by a more detailed characterization of crashes involving 16 t+ trucks based on national road crash data from Italy, Spain, and Sweden. This analysis is further refined by a detailed study of crashes involving 16 t+ trucks in the German In-Depth Accident Study (GIDAS), including a crash causation analysis. The results show that most European HGV crashes occur in clear weather, during daylight, on dry roads, outside city limits, and on nonhighway roads. Three main scenarios for 16 t+ trucks are characterized in-depth: rear-end crashes in which the truck is the striking partner, conflicts during right turn maneuvers of the truck with a cyclist riding alongside, and pedestrians crossing the road in front of the truck. Among truck-related crash causes, information admission failures (e.g., distraction) were the main crash causation factor in 72% of cases in the rear-end striking scenario while information access problems (e.g., blind spots) were present for 72% of cases in the cyclist scenario and 75% of cases in the pedestrian scenario. The three levels of data analysis used in this paper give a deeper understanding of European HGV crashes, in terms of the most common crash characteristics on EU level and very detailed descriptions of both kinematic parameters and crash causation factors for the above scenarios. The results thereby provide both a global overview and sufficient depth of analysis of the most relevant cases and aid safety system development.

## 1. Introduction

In 2019, around 1 million crashes happened on European roads, resulting in 22,995 traffic fatalities [1]. Although fatality numbers in crashes involving heavy goods vehicles (HGV) fell by almost 40% in the EU since 2007 [2], the remaining crashes still have severe consequences. 

While previous studies identified decreasing rates in fatality numbers during the last years [3], this decrease is lower for pedestrians (39%) and cyclists (27%) than for vehicle occupants (44%) or powered two-wheelers (57%). The development and implementation of safety systems in HGVs, in particular active safety systems, can help to reduce the number of crashes even further. The basis for the design of these systems is a deep understanding of HGV-involved crashes and the underlying mechanisms, as well as factors influencing crash causation and outcome. The newly released UN regulations No 151 and 159 outline uniform requirements for a Blind Spot Information System for the Detection of Bicycles [4] and a Moving Off Information System for the Detection of Pedestrians and Cyclists [5] in specific situations to improve the situation for vulnerable road users (VRUs) further. However, these systems are only required to inform the driver (and do not intervene themselves), but no information is available on how drivers behave in these situations where the systems would be active. It is therefore necessary to understand the most critical crash scenarios in more detail and to also analyze the factors influencing the crash causation, to support the design of intervention systems that can mitigate these types of crashes.

This paper gives an overview of currently available studies for HGV-related crashes. It describes the different databases that were used for the analysis and outlines the results obtained. The paper is rounded up by a discussion of the results and limitations and provides an outlook into future research.

## 2. Literature Review

The review of existing literature has revealed only sparse information available for crashes involving heavy goods vehicles in Europe. The technical report by Kockum et al. indicated that HGV occupants in Europe are injured in 10–20% of HGV crashes, while the corresponding figures are 50–55% for car occupants and 30–35% for VRUs, supporting previous findings that car occupants and VRUs comprise the largest group of casualties in truck related crashes also in Europe [6]. 

Other published studies of HGV related crashes focus mainly on USA data. Woodroofe and Blower identified in a study that rollover and head-on collisions are the main collision types for truck driver injuries, accounting for 73% of serious and fatal injuries of truck drivers [7]. However, as shown in Zhu and Srinivasan based on data from the Large Truck Crash Causation study (LTCCS) in the USA, fatally injured persons in crashes that involve heavy trucks are usually occupants of the crash opponent vehicles, and the most serious crash types in terms of the overall injury outcome are head-on collisions and collisions at intersections [8]. Vulnerable road users, lacking a protective shell around them (e.g., crumple zone, airbag), may be especially exposed in crashes with HGV involvement. Kim, Kim, Ulfarsson, and Porrello correlate the involvement of a truck in the crash with a significant increase in the likelihood of a fatal injury of cyclists in the USA [9]. Lee and Abdel–Aty found in an analysis of data from Florida (USA) that the larger size of HGVs was correlated to an increased likelihood of severe injuries for pedestrians at intersections [10]. 

Adminaite, Allsop, and Jost describe crashes between trucks and VRUs especially problematic due to the vehicle size and difference in mass and indicate that the main reason for these crashes is the problematic field of view for truck drivers, making VRUs particularly prone to be in the blind spot and overseen by the truck driver [11]. Seiniger, Gail, and Schreck identified that the development of new safety systems of trucks for cyclist protection should be focused on right turning maneuvers and propose a test methodology to validate new active safety systems [12].

Overall, the literature review of results for crashes involving heavy trucks has revealed various limitations and seemingly contradictory results, especially regarding the study of crash causation. Rezapour, Wulff, and Ksaibati analyzed transport data from Wyoming (USA) and conducted a violation analysis. The authors conclude that in more than 80% of all crashes involving a truck, the truck drivers are the party at fault, which emphasizes the need to introduce more safety systems into trucks [13]. Findings based on European in-depth crash data suggest however that truck drivers are the party at fault in only 25% of cases [14]. The large difference in the estimates underlines the need for further studies. While accident causation results are presented in Evgenikos et al. based on data collected between 2005 and 2008 in the SafetyNet project [15], safety system development may require further analysis based on more recent data and inclusion of precrash information (e.g., trajectories, initial speeds, environment conditions). 

A further limitation to using these data for the design of active safety systems for trucks in Europe is that most studies are based on data sets from North America. Wang and Wei showed in their analysis that benefits achieved by active safety systems in one country cannot easily be transferred to other countries [16], emphasizing the need to analyze regional data. Due to different road infrastructure designs (e.g., wider lanes) and vehicle designs (e.g., conventional cab design in North America compared to flat nose design in Europe), the representativeness of study results based on USA data to the situation in Europe are limited. In 2008, Knight et al. identified a lack of robust European crash data especially for large trucks [17]. Several characteristics of HGV crashes in Europe were then studied in Evgenikos et al., addressing all heavy goods vehicles (over 3.5 t maximum permissible gross weight) [15]. However, there are significant differences in vehicle types in this category that could range from vans like a Mercedes Sprinter to long-haul truck-trailer combinations such as the Volvo FH. Different types of HGVs have different characteristics (e.g., vehicle dynamics, field of view), and a more detailed classification would be important for a more directed safety system development. There are studies on more specific crash scenarios for HGVs available (e.g., [18,19]), but the frequency of these scenarios in Europe is not quantified, and therefore the relevance of these scenarios for European trucks remains unclear.

Therefore, the aim of this paper is to provide descriptive statistics that are based on a comprehensive and up-to-date analysis of HGV crashes in Europe, focusing on heavy long-haul trucks with a combination weight above 16 t (further referred to as 16 t+ trucks).

## 3. Materials and Methods

**Figure 1:**
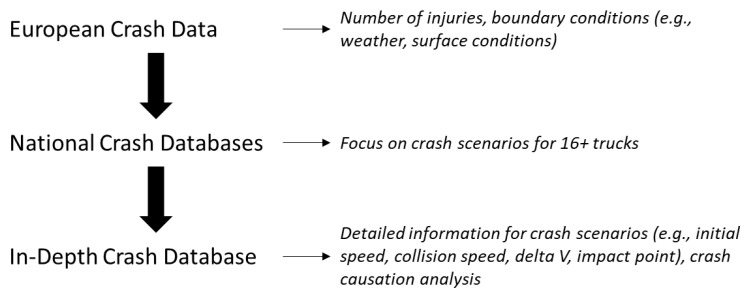
Overview of analysis approach.

The approach to identify relevant crash scenarios consists of three levels (see Figure 1). First, data from the Community Database on Accidents on the Roads in Europe (CARE) is extracted to get a general understanding of crashes with the involvement of heavy goods vehicles in Europe. Since no information on the weight of the involved vehicles is available, this analysis is limited to the vehicle categories coded in CARE, resulting in the inclusion of all crashes that involve a vehicle with a gross weight above 3.5 t. The main goal of this level of the analysis is to obtain representative information on the distribution of injuries and boundary conditions (e.g., weather, road surface condition). As CARE contains only high-level data, this first level of the analysis needs to be complemented by other data sources for the identification and description of relevant crash scenarios.

More detailed information can be obtained from national crash databases, which provides the second level of the analysis. These databases usually contain more information, such as vehicle weight, that allow for a filtering of crashes to those with the involvement of a 16 t+ truck. In this analysis, access to national crash databases from Sweden, Spain, and Italy could be obtained, hence these specific databases were used for the analysis. The focus for these databases was the identification of common critical scenarios and a comparison of injury distributions and boundary conditions to the results from CARE.

**Figure 2:**
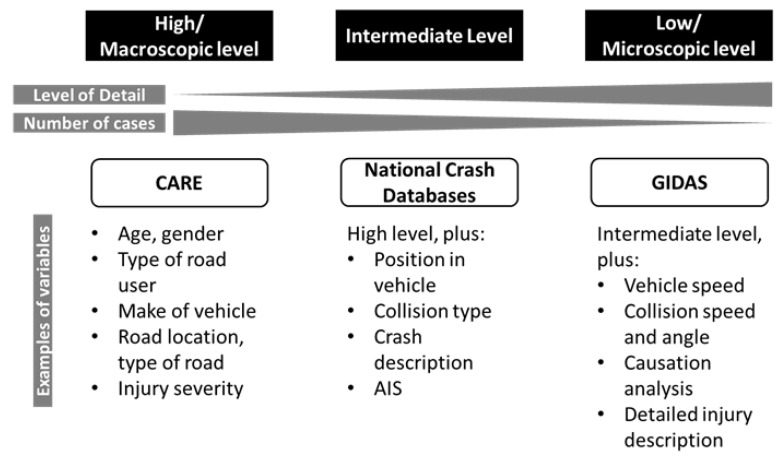
Overview of databases used for analysis.

The third level of analysis provides more detailed information for the critical scenarios identified in the previous level, such as initial speed, collision speed, delta *v*, and impact points. This level is based on in-depth crash data from the German In-Depth Accident Study (GIDAS). In-depth crash databases contain detailed crash scenario descriptions, crash reconstructions and information about causation factors. At the same time, their sampling region and case count are substantially smaller compared to CARE or the national databases. Consequently, each of the three levels of the analysis provides information that complements results of the other two (see Figure 2) and a full picture can only be obtained by their combined analysis.

The injury levels of all persons involved in a crash are based on police-reported crash severity levels, that are defined in the databases as follows:

- Fatal crash: a crash in which at least one person was fatally injured (the person died from the crash within 30 days).
- Serious crash: a crash in which at least one person was seriously injured (hospitalized for at least 24 h), and nobody was fatally injured.
- Slight crash: a crash in which at least one person was seriously injured (hospitalized for less than 24 h or not hospitalized), and nobody was fatally or seriously injured.

Additionally, the severity level of KSI (killed or seriously injured), which is the union of fatal and serious injuries, will be used for the analysis.

The following sections provide more detailed information on the specific databases used, references to the databases and information on how the data of these sources was handled for the analysis.

### 3.1. CARE

As indicated above, EU crash data on an aggregated level are available in CARE. This database is used to obtain general estimates from police-reported crash data from all EU member states and additionally from Iceland, Liechtenstein, Norway, and Switzerland, and the UK [20]. The set of variables included in CARE is specified in the CaDaS glossary [21]. As vehicle weight is not included as a variable, all results based on CARE queries will be using the general definition of HGVs, i.e., with a total weight of 3.5 t or greater.

Data from 2010 to 2015 from EU28 (defined as the 28 EU countries in 2018) were used for this analysis. Police-reported injury severity levels as defined in the previous section are considered for the analysis. Italy, Finland, and Estonia do not distinguish between serious and slight injuries, but rather report a generic number of injured persons. Therefore, for these countries, it was assumed that 14% of all reported nonfatal injuries were serious and 86% were slight, based on a study of Italian data (that gives the large majority of cases; see the next section).

### 3.2. National Crash Databases

In the second stage of the analysis, the national crash databases of Sweden [22], Spain [23], and Italy [24] were analyzed. Different timespans were available for the analysis for each database: Swedish data are based on the years 2000 to 2016, Spanish data are based on the years 2014 to 2016 and Italian data are based on the years 2010 to 2016. All these databases were queried for crashes involving heavy goods vehicles with a gross weight above 16 t.

Since Italian data do not provide a distinction between serious and slight injuries, the reported numbers of nonfatally injured persons were distributed to slightly and seriously injured according to a study by the Italian road infrastructure administration [25], with 86% of the injuries being recoded as slight and the remaining 14% as serious. Additionally, Italian data did not include information about light conditions, hence the corresponding analysis will be given for the other two countries only.

To enable a comparison of crash scenarios for crashes with 16 t+ trucks between the three countries, the corresponding data in the three databases had to be recoded to a common coding scheme. Specifically, the Italian and Spanish databases were recoded to the Swedish crash type classification.

### 3.3. GIDAS

The third level of analysis addressed in-depth crash data involving 16 t+ trucks from the German In-Depth Accident Study (GIDAS). GIDAS is the largest in-depth road crash study in Germany, currently including about 30,000 crashes from the areas of Hannover and Dresden. The crash investigation teams are notified by the police and go on-scene to crashes with at least one injured person. Up to 3000 variables are recorded per crash, including technical vehicle data, crash information, road design, active and passive safety systems, crash scene details and causes of the crash. Following the data collection, each crash is reconstructed to obtain information on crash kinematics and sequence of events. Due to a carefully defined statistical sampling process, the collected crashes are suitable for representing the German crash situation [26].

The crash types developed by the German insurance association GDV [27], which is also used by the police in Germany, serves as a crash classification system for this study and provides more detailed information than the crash types in the national crash databases analyzed before. For the analysis, the opponent involved in the most serious collision was chosen as the collision partner of the trucks for the following analysis, to identify the collision partner associated with the most serious injuries.

**Figure 3:**
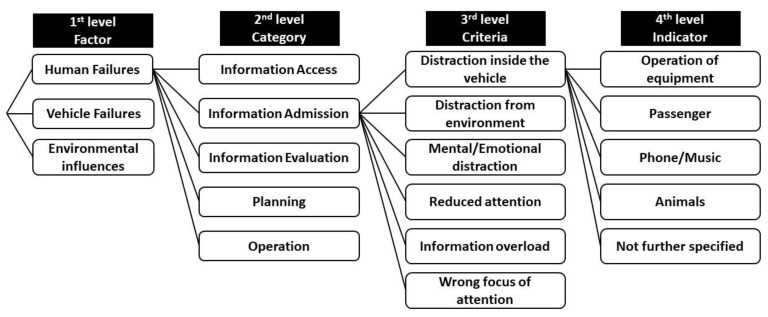
Example of Accident Causation Analysis System (ACAS) classification in German In-Depth Accident Study (GIDAS).

The causes of the crashes are identified using the Accident Causation Analysis System (ACAS) [28], which is based on a structured interview of the involved parties or witnesses. If no interview is possible, the information is collected from police reports or expert opinion of the crash investigators. Three main groups of causation factors are considered: vehicle-based failures (technical defects, illegal technical alterations, or HMI problems), environment-based factors (road infrastructure failures, weather, and other external influences), and human failures. These different levels can be further broken down and specified for the analysis, see Figure 3 for an example. If relevant, multiple causation factors can be assigned to each party involved in the crash. Participants who did not cause the crash (as deemed by the police and investigators) are not assigned with a causation factor. Because ACAS is only available from the Hannover part of the GIDAS data from the years 2008 to 2017, only a reduced number of crashes is available for the crash causation analysis.

## 4. Results

This section describes the results from all three analysis levels. For the percentages reported in this section, injuries and categorical factors that were coded as “other” or “unknown” were excluded, if not indicated otherwise.

### 4.1. CARE

**Figure 4:**
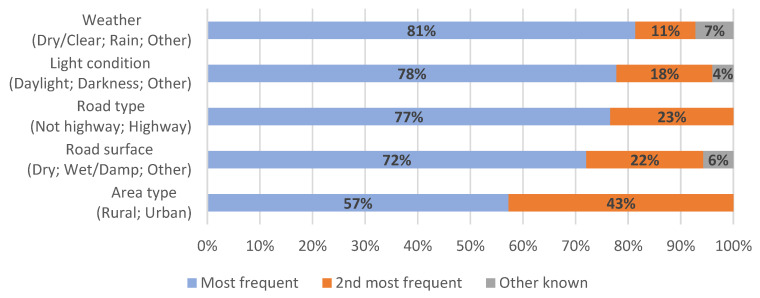
Characterization of crashes involving heavy goods vehicles (HGVs) in Europe in terms of environmental variables, based on Community Database on Accidents on the Roads in Europe (CARE) data.

The high-level analysis based on CARE data shows that HGVs were involved in 4.5% of all crashes and 14.2% of fatal crashes in Europe, indicating an overrepresentation of HGV involvement in fatal crashes. Figure 4 gives a general characterization of crashes involving 3.5 t+ trucks in Europe. This figure shows the names of the considered environmental variables, with the most frequent values specified between parentheses, and the prevalence of these values is indicated by the length of the bars. For example, for the variable “Weather” (code A-6 in CaDaS), the most common value is “Dry/clear” which is present in 81% of crashes involving HGVs in EU-28, followed by “Rain”, present in 11% of crashes, and other values of the variable, present in 7% of the crashes. The results indicate that most crashes involving 3.5 t+ trucks in EU-28 occur in dry/clear weather (81%), daylight (78%), on roads that are not highways (77%), on roads with a dry surface (72%) and in rural areas (57%).

The analogous results for crashes with a serious or fatal outcome show that KSI crashes involving HGVs in EU-28 can be characterized by dry/clear weather (82%), nonhighway roads (77%), daylight (73%), dry road surface (72%) and rural environment (65%). In other words, the results are similar to those for all injury crashes, but with greater percentages of rural crashes (65% vs. 57%) and darkness (22% vs. 18%).

**Figure 5:**
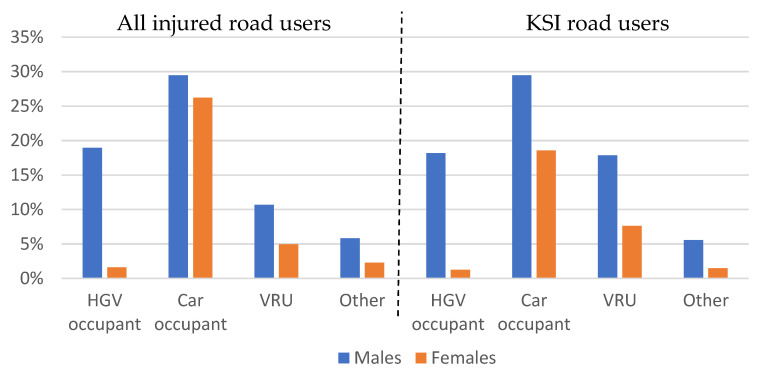
Joint distribution of gender and road user group for people injured in crashes with HGV involvement in EU-28, separately for all injury levels and for KSI, based on CARE data.

Table 1 shows the distribution of injured road users by road user type and age group (both as provided in CARE). Table 1 shows that people injured in HGV crashes in EU-28 are mainly car occupants (55%), followed by HGV occupants (21%), vulnerable road users (16%) and other road users (8%). In more detail, the largest group of injured people in HGV crashes are car occupants and HGV occupants in the age group between 25 and 64 years. This age group is especially prevalent for HGV occupants, as 85% of all injured HGV occupants and 86% of all KSI HGV occupants are in this group. Notably, the percentage of young (<25 years) and old (>64 years) VRUs is much higher among KSI road users (lower part of Table 1) compared to all injury levels (upper part in Table 1). The gender distribution, provided in Figure 5 below, indicates that males are more frequently injured in crashes with HGV involvement than females, with males having a total share of 65% of all injuries and 71% of KSI injuries. The gender distribution is close to equal for injured car occupants and very skewed towards males for injured HGV occupants (92% of whom are males).

### 4.2. National Crash Databases

The databases in Sweden, Spain, and Italy for the analyzed years as specified in the Methods section contained 7069, 5232, and 27,008 crashes with 16 t+ trucks respectively out of which 1569 (22%), 1246 (24%), and 5237 (19%) were in the category KSI. For weather and light conditions, the national crash statistics for 16t+ trucks from the analyzed countries (Sweden, Spain, Italy) generally follow the trends observed for 3.5 t+ trucks in CARE: crashes occurred mostly in dry/clear weather conditions (SWE 77%, ESP 88%, ITA 76%) and in daylight (SWE 73%, ESP 74%, ITA n.a.). Bigger differences between countries (and towards CARE) can be observed for surface conditions, where most crashes with 16t+ trucks occur with dry surfaces (SWE 51%, ESP 83%, ITA 81%), on non-highway roads (SWE 81%, ESP 54%, ITA 69%) and in rural areas (SWE 60%, ESP 87%, ITA 66%).

**Figure 6:**
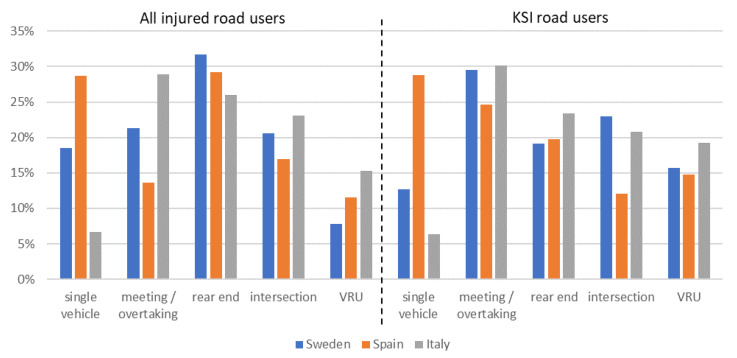
Distribution of crash scenarios for people injured in crashes with HGV involvement, separately for all injury levels and for KSI road users, based on the respective national crash databases.

Crash type distributions can be compared across countries after recoding as described in the Materials and Methods section, see Figure 6. The figure shows the importance of rear-end crashes among all injury crashes and a greater prevalence of VRU crashes and meeting/overtaking crashes among KSI crashes compared to that of their shares among all crashes, indicating more serious injury outcomes in these crash types.

Notable differences between the crash type results for the analyzed three countries include the high proportion of single vehicle crashes in Spain (especially for KSI crashes) while the proportion of meeting/overtaking crashes among all injury crashes and the intersection crashes among KSI crashes is smaller in Spain compared to Italy and Sweden.

### 4.3. GIDAS

The GIDAS database contains 1091 16 t+ trucks that were involved in a crash between the years 2000–2017. The majority of analyzed crashes happened during daytime (75%) and occurred outside city limits (59%) and on motorways (42%). Most 16 t+ trucks had a crash outside of junctions, either on a straight stretch of road (55%) or in a curve (9%). Crashes at junctions (11%) or crossings (20%) were less frequent.

**Figure 7:**
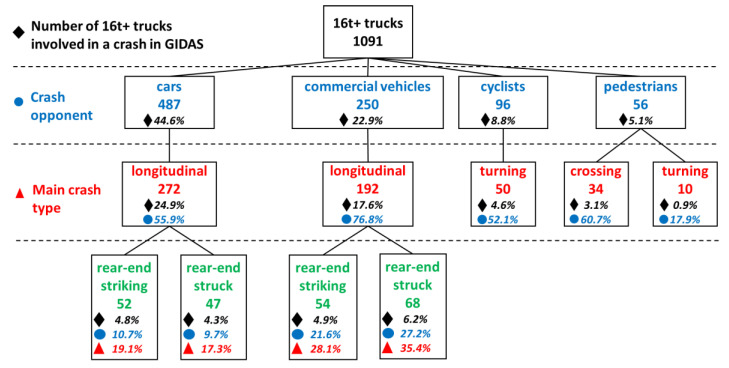
Most common crash types from GIDAS (case count and percentages per category); less frequent crash types are omitted for readability.

For the analysis, 165 cases with unknown accident type or unknown collision partner as well as single vehicle crashes were discarded. Table 2 shows a summary of the different collision partners of 16 t+ trucks and their involvement in the different crash types. Sixteen t+ trucks are mostly involved in crashes with cars (487 cases, 44.6%), the majority of them occurring due to a conflict in longitudinal traffic (in 272 cases (55.9% of truck-to-car cases)). Figure 7 gives an overview of the most common scenarios in the GIDAS database and shows that 35.7% of crashes are rear-end collisions (in which the truck was the striking vehicle in 54% of cases) and 23.9% occurred due to a lane-change maneuver (in which the truck changed lane in 63% of cases).

Commercial vehicles (buses and HGVs) are the second most frequent crash opponents of 16 t+ trucks (22.9%). Crashes in longitudinal traffic are again the most frequent types (76.8%, corresponding to 17.6% of all cases). In 28.1% of longitudinal cases, the 16 t+ truck is the striking vehicle in a rear-end collision, and in 35.4% the struck vehicle; crashes between two 16 t+ trucks are only counted in the rear-end striking category to avoid double-counting. In contrast to accidents with cars, lane change accidents rarely occurred between a heavy truck and another commercial vehicle.

Among the vulnerable road users, cyclists have the highest share as crash opponents for 16 t+ trucks. The most common specific accident type with 44.8% is when a truck turns right and has a conflict with a cyclist travelling alongside in the same direction mostly on a bicycle path on the right side of the road.

Conflicts between 16 t+ trucks and pedestrians were found in 5.1% of all cases. The most frequent category among those crashes is when a pedestrian enters the road to cut across perpendicular to the direction of travel of the truck in 60.7% of cases. The second most frequent category of crashes with pedestrians is when the truck turned off the main road and had a conflict with a pedestrian walking on the sidewalk (17.9%).

Crashes of 16 t+ trucks with powered two wheelers were not as common as with other road users (3.4% of cases). These cases were mostly from the accident type categories of “turning off accidents” or “accidents in longitudinal traffic” and are not further investigated due to the low number of cases.

### 4.4. Definition and Description of Critical Scenarios

Based on the most common crash types and results from CARE and national crash databases, three crash scenarios for 16 t+ trucks were established as most relevant to further investigate in a more detailed crash analysis. These scenarios are in line with the focus on the most common crash scenarios as well as VRU-involved crashes,

1. Scenario 1: rear-end crashes with cars and commercial vehicles as collision partners. Due to the focus on preventability of the crash truck-based safety systems, only cases where the 16 t+ truck is the striking vehicle are considered for further analysis.
2. Scenario 2: conflicts between a truck that is turning right and a cyclist that is travelling alongside with the intention to go straight.
3. Scenario 3: conflicts with a pedestrian crossing the road in front of the truck.

These scenarios address the three different road user types, with scenario 1 as the overall most frequent one and scenarios 2 and 3 as the most frequent crash scenarios with VRUs as the crash opponent. The focus on VRU crashes is motivated by the high crash severity outcome in these crashes indicated by previous literature results detailed in the introduction as well as Figure 6 and is in line with the EC objective number 7 on improved protection for cyclists and pedestrians (EC, 2010).

#### 4.4.1. Scenario 1: Rear-End Crashes, 16 t+ Truck as Striking Vehicle

**Figure 8:**
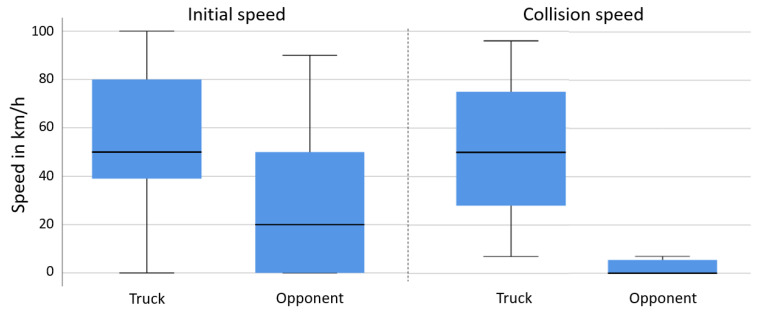
Scenario 1, initial and collision speeds of trucks and collision partners in rear end crashes, based on GIDAS.

In the first scenario, including 106 cases from the GIDAS database (11.4% of all cases), the median travelling speed of the trucks at conflict initiation was 50 km/h, with 25% of the trucks travelling at speeds above 80 km/h (see the box plot in Figure 8). The initial speeds of the struck vehicles were substantially lower at the initiation of the conflict (median: 20 km/h). Collision speeds were lower than the initial travelling speeds for both vehicles, with most collision opponents standing at the time of collision.

**Figure 9:**
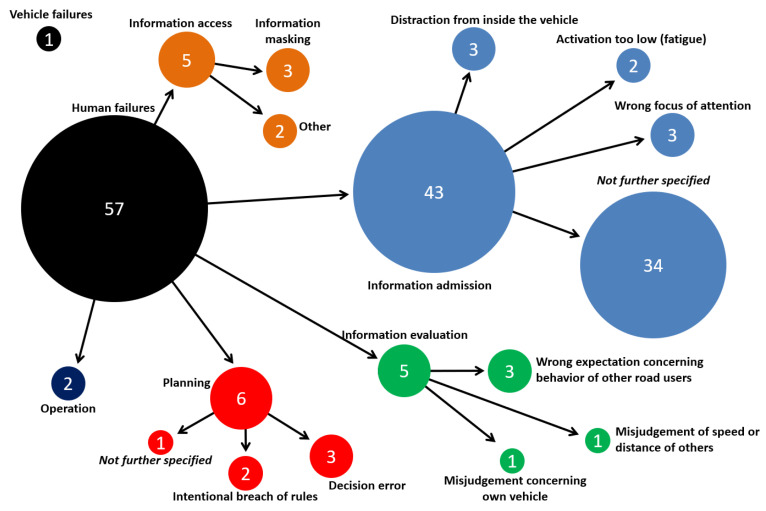
ACAS analysis of truck drivers in scenario 1 (rear-end striking crashes).

For this scenario, 60 cases are available with Accident Causation Analysis System (ACAS) coding in the GIDAS database (see Figure 9) of which 57 were assigned a human failure, one case included a vehicle failure (brakes) and in 2 cases, the truck had not caused the crash. A total of 75% of human failure cases and thereby 72% of the cases in this scenario were from the category of information admission. These were mostly not further specified as the drivers of the trucks often could not be interviewed or did not remember the crash due to its severity. Less frequent human failure categories were information access problems (8.8%), information evaluation failures (8.8%) and planning failures (10.5%).

#### 4.4.2. Scenario 2: Right-Turn Conflicts with Cyclists

**Figure 10:**
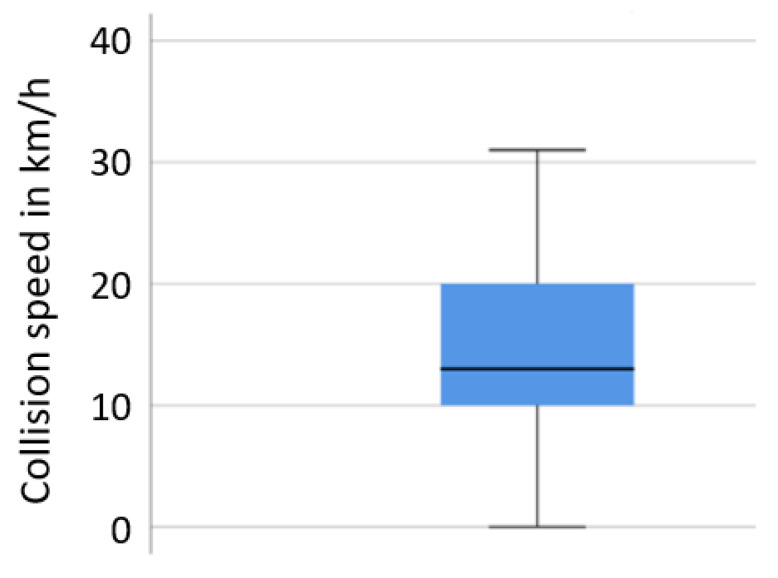
Scenario 2, collision speed of truck in right turn crashes, based on GIDAS.

The second scenario includes right turning trucks that had a conflict with a cyclist travelling along the initial direction of the truck in 43 cases (4% of all cases with 16 t+ trucks in GIDAS). The conflicts occurred at lower collision speeds of the truck compared with scenario 1, with a median of 13 km/h (see Figure 10). No reliable speed data are available for the cyclist.

**Figure 11:**
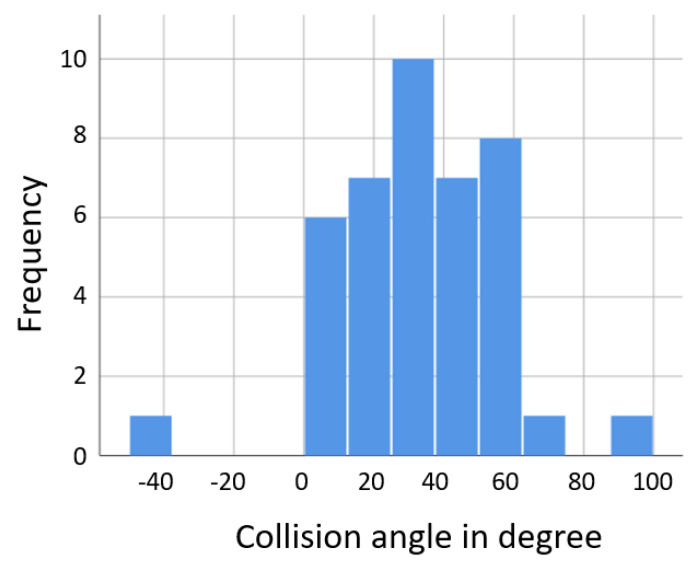
Scenario 2: collision angles between truck and cyclist in right turn crashes, based on GIDAS.

**Figure 12:**
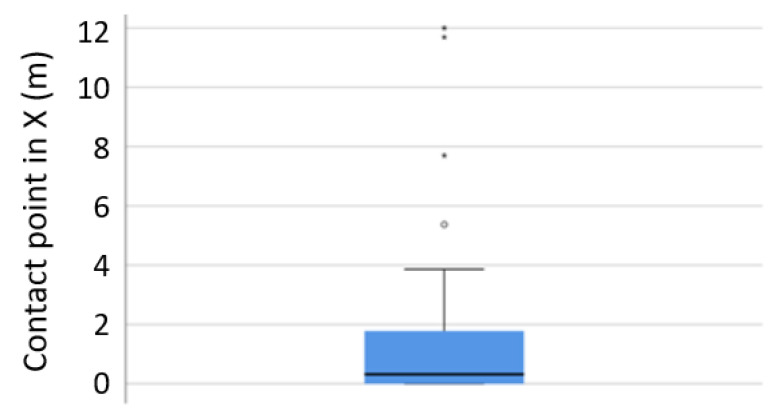
Scenario 2: contact point of cyclist with truck in x-direction from front of truck, based on GIDAS.

The collision angle between cyclist and truck describes the angle between the motion vectors of the truck and cyclist at the point of the collision. It was found to be between 0° and 60° in most cases with a peak at 30° (see Figure 11). In 75% of the cases, the cyclist collided with the side of the truck within the first 2 m from the front of the truck, see Figure 12. Only 4 contact points had higher values than 5 m (i.e., were behind the cabin and further towards the trailer/rear axle of the truck).

**Figure 13:**
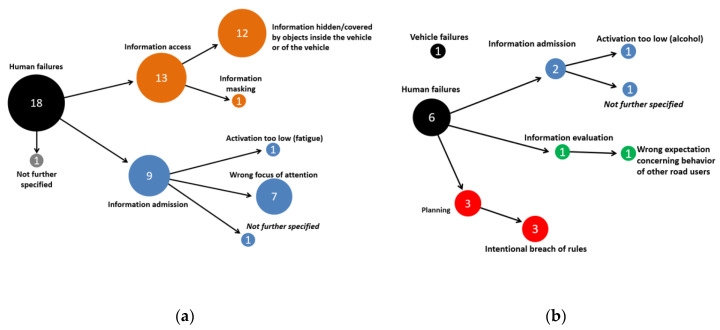
ACAS analysis of truck drivers (**a**) and cyclist (**b**) for Scenario 2 (right-turn crashes).

For this scenario, 18 drivers of 16 t+ trucks were assigned with causation factors, which were all from the group of human failures, see Figure 13a. Of these cases, 72% included information access problems—here, mostly that the relevant information (i.e., the cyclist) was hidden by bodywork of the truck (e.g., the driver’s cabin). For 39% of the 18 truck drivers, a wrong focus of attention such as a missed reassuring view (information admission failures) was reported.

Cyclists were less often identified to cause crashes in Scenario 2 compared to that of the truck drivers (see Figure 13b). There are 6 cases where the cyclist was assigned a human failure (out of which 50% were an intentional breach of rules such as an irregular use of roadway) and one case with a vehicle failure (brakes did not work properly).

#### 4.4.3. Scenario 3: Conflicts with Crossing Pedestrians

**Figure 14:**
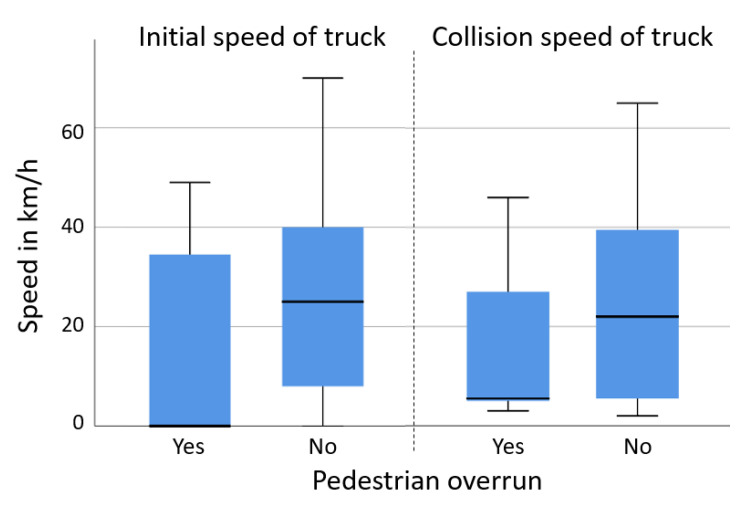
Scenario 3: initial speeds of truck in pedestrian crossing crashes, based on GIDAS.

In the third scenario, including 34 cases (3% of all analyzed GIDAS cases), the pedestrian was overrun by a part of the truck in 16 cases (which leads to more serious or fatal injuries). In 10 out of the 16 cases, the truck was initially standing (e.g., at a traffic light) and the pedestrian crossed the road directly in front of the truck when the truck started to accelerate. Thus, the collision speeds here were under 10 km/h in most cases (see Figure 14). In the remaining cases where the pedestrians were not overrun, the truck had a considerably higher average collision speed at 23 km/h (in 25% of cases above 40 km/h).

The speed at the initiation of the conflict for the cases where the pedestrian was not overrun were similar to the collision speeds (see Figure 14), suggesting little or no braking happened before the collision in many cases.

**Figure 15:**
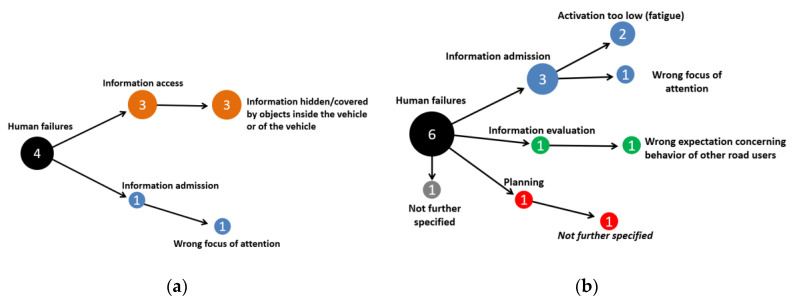
ACAS analysis of truck drivers (**a**) and pedestrians (**b**) in Scenario 3 (pedestrian crossing crashes).

For this scenario, 4 truck drivers and 6 pedestrians were assigned with a human failure code in ACAS (no vehicle or environmental failure was present). Three of the four cases for truck drivers (75%) included information access problems—here, mostly that the relevant information (i.e., the presence of movement of the pedestrian) was hidden by bodywork of the truck (e.g., the driver’s cabin), see Figure 15a. These cases resulted in the pedestrian being overrun by the truck, whereas in the one case left where the pedestrian was not overrun, the truck driver had a wrong focus of attention. There are 6 cases with human failures of the pedestrian (see Figure 15b), out of which 50% are from the information admission. Out of these 6 cases, there was only one case where the pedestrian was not overrun, and it fell into the category “activation too low”.

## 5. Discussion

This study provides an up to date three-level analysis of crashes that involve heavy goods vehicles in the European Union. Our results update and complement previous studies, such as the material used for the preparation of UN regulations No 151 [4] and 159 [5], with a narrower scope on the vehicle type and a wider coverage of different crash databases during the analysis. Three critical crash scenarios were identified and studied in more detail, providing a deep insight into these scenarios.

Compared to that of previous studies in North America, the results obtained in the crash data analysis at hand show similar results. For example, Zhu and Srinivasan [8] also identified collisions in longitudinal traffic and collisions at intersections as the most common crash types in the USA. Our results also support previous findings by Adminaite, Allsop, and Jost [11] who identified a problematic field of view as a main contributing factor in VRU related crashes. From our analysis, HGVs showed an overrepresentation in fatal crashes, supporting previous findings of increased risk of fatal injuries in HGV-involved crashes by Lee and Abdel–Aty [10].

Some of the differences seen between the data from CARE, national databases, and GIDAS can result from the existence of local differences (e.g., there are more days with rain in Sweden compared to that of Spain, resulting in a higher exposure to rainy weather conditions in Sweden), thus a higher exposure to these situations. On the other hand, the differences could also be attributed to different filter criteria between the different databases (i.e., weight restriction to above 3.5 t for CARE and 16t for national databases and GIDAS).

Further uncertainties in the analysis are introduced by the different coding schemes. The different European countries are using different coding schemes, e.g., for crash types. While we have re-coded crash types to a common definition for this analysis, this inherently means a loss in detail. For example, it is no longer possible to talk about detailed crash scenarios and compare those between the different national databases, but a more generic categorization (see for example Figure 6) needs to be used. In addition, there are crash types that are difficult to recode between the different countries, and even at the point of data collection, police officers might have difficulties to categorize the crash consistently. Indicators for the difficulty can be seen in single vehicle crashes where, by definition, only truck occupants should be injured, but also car occupant and VRU injuries are sometimes reported.

Furthermore, we would like to emphasize that the reported results are based on absolute numbers and distributions and have no direct relation to risk. For example, 78% of crashes happening during daylight does not necessarily mean that driving during daylight is riskier than driving at night. There are more trips performed during daylight, which leads to a larger absolute number of crashes, while the risk for a crash (e.g., the number of crashes normalized by distance driven) could still be lower than during nighttime.

## 6. Conclusions

This study provides a deeper understanding of crashes involving 16t+ trucks on European roads by a comprehensive data analysis conducted simultaneously on three levels. The investigation of crash causation provides a deeper insight into contributing factors in crashes involving heavy goods vehicles (HGVs) and provides valuable information for the design of safety systems that are designed to work in these situations.

Safety system developers can use the detailed scenario characterizations to improve safety systems and safety evaluations by focusing on the most relevant crash situations and achieve the highest benefits. In particular, the results from this work were the basis for the further development of active and passive safety systems for heavy, long-haul trucks within the EU H2020 project AEROFLEX. In addition, the results of this work aim to improve the safety of heavy trucks by providing the data input necessary to develop test protocols. Furthermore, the identified scenarios could be the basis for a testing and rating scheme for HGVs, similar to what is implemented by Euro New Car Assessment Programme (NCAP) for passenger cars.

Most crashes that involve HGVs occur in dry and clear weather conditions (76–88%, depending on region), during daylight conditions (73–78%), on dry roads (51–83%), outside city limits (57–87%) and on nonhighway roads (54–81%). All three analysis levels show the same trends regarding these variables, but small differences exist. The reasons for these differences could range from local effects (e.g., weather, driving behavior, vehicle types) to filter criteria in each database (e.g., weight or size restrictions).

As a result of the three-stage analysis, three scenarios were identified that should be addressed by future research and safety systems: (1) rear-end crashes with other vehicles in which the truck is the striking partner, (2) conflicts during right turn maneuvers between the truck and a cyclist travelling alongside with the intention to go straight, and (3) pedestrians crossing the road perpendicular to the direction of travel of the truck. These three scenarios were studied in detail in the GIDAS database, leading to the following conclusions:

1. In rear-end striking crashes, the average speed of the truck at conflict onset is about 50 km/h and by the time of the collision, it is reduced to about 30 km/h, whereas the struck vehicle is typically standing at impact. In 95% of cases, human failures were identified as the causing factor (with information admission identified as the most common category, prevalent in 72% of truck-related causes).
2. During the right turn maneuvers, the average collision speed of the truck is about 13 km/h and the impact happens at an angle of 33 degrees on average, with the impact point within the first 2 m along the length of the truck (i.e., around the area of the passenger-side door). In this scenario, problems with information access (e.g., blind spots) were identified in 72% of cases for the truck drivers, and in 27% of all crashes the cyclist was identified as the party at fault.
3. In the pedestrian crossing scenario, initial truck speeds are generally low (<5 km/h) for cases where the pedestrian is overrun by the truck. Such a case often results from situations when pedestrians cross the road in front of a waiting truck and are not perceived by the truck driver when starting to accelerate. Collision speeds are higher (>20 km/h) when the pedestrians are not overrun. Overall, problems with information access were identified as the main causing factor in 75% of cases for truck drivers. Even though the speeds in the vulnerable road users (VRU)-related scenarios are generally low, the outcomes are serious, especially when the VRUs are overrun by the truck.

## 7. Limitations and Future Recommendations

The interpretation of the conclusions stated in the previous section should consider several limitations that follow from the aspects mentioned in the Discussion. For example, while national datasets analyzed here include countries with a reasonably wide geo-graphical spread across Europe, the analysis of three national datasets may not fully rep-resent crashes with 16 t+ trucks in the EU. Especially for the interpretation of the differences seen between national crash databases and CARE, it would be beneficial to include more national databases in the analysis, to get a better understanding of local differences. To which extent the observed differences are an undesired result of different filter criteria between national databases and CARE or represent actual local differences is therefore recommended for future research.

As argued in the Discussion, to be able to analyze risk, exposure data are needed, which is extremely difficult to obtain. When crash data and exposure data are both avail-able, risks for specific factors (such as daytime or weather) can be calculated and analyzed. Unfortunately, exposure data, especially for specific situations like day/nighttime driving, are very difficult to obtain. To enable conclusions based on risk estimations without access to exposure data, the application of the induced exposure methodology (e.g., [29,30]) could be explored in future research on this topic.

Finally, this paper is dedicated to the analysis and characterization of road crashes which are safety-critical events ending in a collision. Another important step for the development and safety benefit assessment of active safety systems that can potentially help avoiding the collision is the understanding of road user behavior in the conflict situations that precede such crashes, including normal driving situations, as well as critical situations. Based on the analysis results presented in the previous sections, authors of this paper conducted an experiment for the VRU-related situations. The results of the analysis at hand were used to replicate the conflict situations in a controlled test-track experiment with professional truck drivers (see [31]). The data of this experimental study are used for further driver behavior analysis (see [32]) that can supplement the insights gained by the crash data analysis presented in this paper. A research question that could be investigated by future research is to which extent crash causes identified in this paper may be related to observations made in naturalistic driving data.

## Figures and Tables

**Table 1:** Joint distribution of age and road user group for people injured in crashes with HGV involvement in EU-28, separately for all injury levels and killed or seriously injured (KSI), with rounded values, based on CARE data.

		<18	18–24	25–64	>64	TOTAL
All injured road users	HGV occupant	0%	2%	18%	1%	21%
	Car occupant	4%	9%	36%	6%	55%
	VRU	2%	2%	9%	3%	16%
	Other	1%	1%	5%	1%	8%
	TOTAL (*n* = 462,107)	7%	14%	69%	10%	100%
KSI road users	HGV occupant	0%	2%	17%	1%	20%
	Car occupant	3%	8%	30%	8%	48%
	VRU	3%	3%	14%	6%	25%
	Other	1%	1%	5%	1%	8%
	TOTAL (*n* = 109,825)	6%	13%	66%	16%	100%

**Table 2:** Categories of crash types for 16 t+ trucks with different types of road users as crash opponents, based on GIDAS.

Categories Based on Initial Conflict	Cars	Commercial Vehicles	Bicycles	Pedestrian	Powered Two-Wheeler	TOTAL
1 Driving accident	39	12	3	0	3	57
2 Turning off accident	44	11	50	10	10	125
3 Crossing/entering accident	81	15	30	0	7	133
4 Pedestrian crossing road	1	0	0	34	0	35
5 Accident with parked vehicle	20	8	4	2	3	37
6 Accident in longitudinal traffic	272	192	8	2	14	488
7 Other accident types	30	12	1	8	0	51
TOTAL	487	250	96	56	37	926

## Data Availability

The data used for this analysis are not owned by the authors or their institutions and are not publicly available. More information about each analyzed dataset is available in the corresponding references below.

## Abbreviations

16 t+ truck	Truck with a combination weight above 16t
ACAS	Accident Causation Analysis System
CARE	Community Database on Accidents on the Roads in Europe
GIDAS	German In-Depth Accident Study
HGV	Heavy Goods Vehicle (with a weight above 3.5t
HMI	human-machine-interface
KSI	killed or severely injured
LTCCS	Large Truck Crash Causation Study (USA)
STRADA	Swedish National Crash Database
VRU	Vulnerable Road User (e.g., cyclist, pedestrian)
